# Rapid-Onset Obsessive-Compulsive Disorder With Hallucinations in a Post-seizure Four-Year-Old Male

**DOI:** 10.7759/cureus.62630

**Published:** 2024-06-18

**Authors:** David Lopez-Canelas, Lourdes J Delgado-Serrano

**Affiliations:** 1 Pediatric Psychiatry, University of Illinois College of Medicine Peoria, Peoria, USA

**Keywords:** rapid ocd exacerbation, early childhood ocd, pans, pandas and ocd, hallucinations, post seizure, rapid onset ocd, pediatric ocd

## Abstract

Rapid-onset obsessive-compulsive disorder (OCD) has been classically described in the context of infectious and autoimmune stressors, most famously PANDAS (pediatric autoimmune neuropsychiatric disorders associated with streptococcal infections) and then PANS (pediatric autoimmune neuropsychiatric syndrome). PANS itself, however, specifically excludes neurological and medical disorders, including seizures, from the diagnostic criteria. Changes in affect, such as depression/anxiety and new-onset psychosis, have been previously described in the post-seizure period but often self-resolve. To the best of our knowledge, neither rapid onset nor exacerbation of OCD have been previously reported in a post-seizure patient. We present the case of a four-year five-month-old male with a history of poor weight gain who presented to the emergency department for a seizure in the context of hypoglycemia. During the hospital course and within one month following discharge, he became significant for a myriad of new behaviors, rituals, and even visual hallucinations. We propose that the seizure itself is a highly unique and likely neurophysiological stressor. We consider neurologically exacerbated OCD to be an area ripe for further investigation.

## Introduction

Obsessive-compulsive disorder (OCD) is defined as either the presence of obsessions, compulsions, or both [1]. In terms of prevalence, OCD accounts for 1-2% of the global population in both adult and pediatric populations [1,2]. OCD can be divided into early-onset and adult presentations [2]. Within the early onset, which makes up the majority of cases, the mean age of diagnosis was noted at 10.3 years [3]. Furthermore, OCD in younger children is not unheard of, with one study citing a mean age for younger children of 4.95 years at the time of diagnosis [4]. Indeed, prepubertal onset cases account for up to 30% of all OCD presentations [2]. However, it must be remembered that OC (obsessive-compulsive) symptoms will often precede a formal diagnosis in an average of two to five years [4,5]. In terms of heritability, early-onset OCD has an association of 10-fold among first-degree relatives [2].

Onset and presentation are commonly gradual and can be attributable to various psychosocial stressors or trauma [2]. When the presentation is rapid, autoantibodies associated with GABHS (group A beta-hemolytic Streptococcus) and other autoimmune workups are routinely performed. In the absence of streptococcal antibodies, PANDAS (pediatric autoimmune neuropsychiatric disorders associated with streptococcal infections) can be ruled out. Furthermore, in the absence of other neurological or medical disorders, a diagnosis of PANS (pediatric acute-onset neuropsychiatric syndrome) is reasonable [6]. 

In terms of symptoms, pediatric and adult populations can differ, with compulsions only being more prominent in early-onset OCD [2,7]. In general, symptoms can consist of obsessions, including intrusive thoughts, or compulsions, including ritualized behaviors, that have a distressing impact on the patient in daily life. In OCD in very young children, patients are often unable to clearly articulate their thoughts or explain their actions, which makes the diagnosis even more challenging [4]. Thus, parent/family recall becomes crucial. The family history may contribute to why compulsive rituals are more observable than obsessive thoughts or worries in pediatric patients. Additionally, the ability to articulate false perceptions is also extremely limited in very young patients [2]. Finally, family accommodation of the patient's worries and rituals can have a deleterious effect on the prognosis [7].

In this study, we discuss the case of a four-year five-month-old patient with an initial diagnosis of OCD and visual hallucinations one month following admission for hypoglycemic seizures. Other comorbidities, including tics, anxiety, and depression, became readily apparent over time. We follow him mainly through his first two years. At the time of writing this article, the patient is currently an adolescent, approximately 14 years old. Even so, his unique historical presentation may contribute to the scant existing literature on OCD in the context of neurological injury.

## Case presentation

At the time of presentation to the emergency department, the patient was a four-year five-month-old male with a chronic history of poor weight gain who had a seizure-like episode in the home lasting 30 minutes in which he was notable for bilateral arm flexion, generalized stiffening, and vertical deviation of the eyes. Following this episode, he became somnolent and lethargic. In the emergency department, the patient was found to have a blood glucose of less than 20, an elevated white count of 18.24, and elevated urine ketones. Table 1 shows in-patient labs. Throughout the admission, the patient was consistently afebrile, with no infectious symptoms or sick contacts; the 24-hour EEG was also within normal limits. Head CT was furthermore unremarkable, as were brain MRIs in subsequent months. A diagnosis of hypoglycemic seizures in the context of inadequate food intake was made. Endocrine and pancreatic lab values were also normal. Following glucose resuscitation, scheduled accu-checks, and IV fluid resuscitation for eight hours, the patient was subsequently discharged after two days with a normal white count and glucose monitoring. Medications at discharge included acetaminophen for pain and cyproheptadine (started prior to admission) for appetite stimulation. Of note, the patient had a g-tube (gastronomy tube) placed by gastroenterology within six months following the seizure for repeated inadequate caloric intake. Even up to the present, the patient continues to depend on the g-tube to supplement per oral caloric intake. Referrals were made for neurology and psychiatry. Within one month, the patient was diagnosed with epilepsy and inattention spells by pediatric neurology. He was started on antiepileptic therapy, including levetiracetam titrated up to 10 mg/kg. However, the patient stopped levetiracetam after the first few months because of emerging rashes. After the first year, he was started on zonisamide alone, titrated up to 160 mg nightly. Repeat yearly EEGs were consistently negative, and no repeat seizures or spells were reported after five seizure-like episodes and inattentive spells in the first year. The patient later stopped antiepileptic therapy five years after the initial seizure and has been seizure-free ever since.

**Table 1:** Relevant objective values during admission for new-onset seizure Values in *bold* are abnormal. WBC: white blood cell, RBC: red blood cell, HGB: hemoglobin, HCT: hematocrit, MCV: mean corpuscular volume, MCH: mean corpuscular hemoglobin, MCHC: mean corpuscular hemoglobin concentration, RDW: red cell distribution width, MPV: mean platelet volume

Labs during admission	Patient value	Reference range
WBC	18.24	5.10–13.40 × 10^3^/mcl
RBC	4.59	3.89–4.97 × 10^6^/mcl
HGB	12.8	10.2–12.7 g/dl
HCT	36.67	31.0–37.7%
MCV	80	71.3–84.0 fl
MCH	27.9	23.7–28.3 pg
MCHC	34.9	32.0–34.7 g/dl
Platelets	341	202–403 × 10^3^/mcl
RDW	12.5	12.5–14.9%
MPV	8.6	9.0–10.9 fl
Neutrophils	80.5	34.0–79.0%
Lymphocytes	12.3	11.0–50.0%
Monocytes	7.1	3.0–14.0%
Eosinophils	0	0.0–2.0%
Basophils	0.1	0.0–1.0%
Absolute neutrophils	14.69	2.00–7.10 × 10^3^/mcl
Absolute lymphocytes	2.24	0.50–4.40 × 10^3^/mcl
Absolute monocytes	1.3	0.30–1.20 × 10^3^/mcl
Absolute eosinophils	0	0.00–0.30 × 10^3^/mcl
Absolute basophils	0.01	0.00–0.10 × 10^3^/mcl
Sodium	135	137–145 mmol/L
Potassium	4.9	3.5–5.1 mmol/L
Chloride	99	98–107 mmol/L
CO_2_, venous	12	22–30 mmol/L
Anion gap	24	<18
BUN	22	9–20 mg/dl
Creatinine	0.53	0.20–0.70 mg/dl
BUN/CR ratio	42	12–20 ratio
Glucose	<20	60–99 mg/dl
C-peptide	1.11	0.78–5.19 ng/mL
IGF1	68	28–241 ng/ml
IGFBP3	2.3	1.0–4.7 mcg/ml
Lactic acid	0.8	0.7–2.1 mmol/L
Urine ketones	3+ (150)	Negative
CT head WO contrast	No acute intracranial pathology	

The patient presented for an initial psychiatric visit five weeks following his hospital admission for a seizure. The mother, who served as the primary historian, had compiled a detailed list of novel and distressing behaviors observable in her son in the months prior, during hospitalization, and in the period up to the first psychiatric visit. Table 2 lists these concerning behaviors. While distressing behaviors had been noted leading up to the seizure, their frequency quickly multiplied following the seizure. Of particular note, within 24 hours of his seizure, the patient started grouping his potato chips into piles that he would eat or discard. He exclaimed that he could see black dots and bugs in his food and would spit out certain foods while mentioning spiders.

**Table 2:** Chronology of new behaviors in patient as observed by mother

Chronology of patient behaviors: mother's observations											
												From infancy			3 months before seizure			Within 1 week following seizure			At 5 weeks (date of psychiatric evaluation)		
Fear of car seat			Refusing to each individual fruit snacks until assigning each a named character and having mom repeat back-named character			Close inspection of food, putting of chips and cereal in piles to eat and not eat. Frequently spitting out food. It takes two hours to eat a bowl of cereal due to the need to inspect individual pieces.			Indecisiveness in eating: Needing to be told at which end to start eating cantaloupe pieces. Needing mom to inspect each bite of food before swallowing.		
When waking from naps, some shaking, odd facial expressions, glazed eyes			Fear of contamination, refusing to play in sandbox, previously often.			Visual hallucinations: seeing bugs and spiders in bowls of food, many foods rejected with perseveration on "that's my spider problem."			Indecisiveness and repeated reassurance seeking: refusing to make choices about where to walk in house given multiple routes, taking up to 45 minutes.		
Separation anxiety			Fear of contamination, becoming upset if shoes ever get dirty or muddy or wet.			Refusing any brown colored foods including chocolate milk previously enjoyed.			When removing shoes needs to be told which to remove first, cannot decide for himself.		
Self blame/increased sensitivity when corrected or accidentally breaking toys			Petting family dog every 20-30 minutes while repeating statement describing his actions.			Insisting on clear cups to see drink contents.			False belief: patient is poisonous, must not be touched and becoming irate if touched, believing he has poisoned others.		
Extreme worry about family members if they become sick, stressed or upset			Refusing food, drink, and bathroom trips except from mom while at home or only with grandpa while at grandparents home			Fear of cups recently taken out of the dishwasher			Can't decide which stairs to use in house without being told		
Sensitive about personal eating utensils becoming upset if they are touched by others			Repeating phrase when self soothing: "I need you to help me not be sad" or "I need you to help me be not upset"			Refuses all pasta except for cup ramen (previously enjoyed various pastas)			Turning ritual with food plate, patient will turn the plate at 1/4 turns asking repeatedly which way plate should be facing. Needing constant reassurance.		
			Speech difficulties: stuttering of fragment sentences and starting sentances over frequently			Constantly pacing and circling when talking			Cannot sit at kitchen table without being told from which side he should take his seat.		
			Needing to restart from the beginning whenever interrupted during speech or activities			Refuses to go to bathroom without having someone outside and the door cracked			New spinning self in a circle while talking in addition to previous pacing and walking in a circle.		
			Ritualized dressing regimen: always pants before shirt and becoming distressed if shirt before pants			increased fear of the dark, bugs, monsters, Halloween, and certain toys.			Cannot tolerate mom leaving home.		
			Indecision: new bathroom added house, would need to be told which bathroom to use since he could not decide for himself.			Fear of leaving the home where he previously enjoyed going out.			Needs validation when taking bites of food, asks mom to inspect his mouth with each bite.		
			Would not permit siblings to help him with his toys, becoming distressed if others touched them.			Repeatedly asking siblings if mom and dad are going to die when not at home.					
						Petting dog frequently, often stopping other activities to go pet dog and even interrupting sleep.					
						Cannot tolerate if mother is not fully dressed, mother in bathrobe, or wearing her reading glasses, stresses out patient.					

By the time of the initial psychiatric visit, the patient was only consuming ramen noodles. He could not tolerate being interrupted during his speech without needing to start over. Furthermore, he would not permit others to touch him. He had also started petting his dog with 20 hand strokes each time every 20-30 minutes and could not bear to be interrupted. New thoughts included, "Am I poisonous?" and seeing spiders, insects, and black dots in his food where others could not.

Although parents declined to have anti-streptococcal antibodies performed, all other autoantibodies, inflammatory markers, and physical exams were negative, making an autoimmune or post-infectious etiology less likely. The family further denied any history consistent with a sore throat or pharyngitis. Genetic studies were also performed, which were noteworthy for the patient being a heterozygous carrier of the L292X variant in the PEX7 gene. This mutation has been associated with pathogenicity when homozygous recessive [8].

With organic, autoimmune, and genetic etiologies having been ruled out, a diagnosis of OCD with poor insight was made. A diagnosis of visual hallucinations was also made based on the patient's history and distress. Neuropsychological evaluation concurred with a diagnosis of OCD and hallucinations while remaining equivocal for autism spectrum disorder.

The family was initially hesitant to begin psychotropic therapy but later agreed to start fluoxetine up to 20 mg daily with limited benefit. Fluoxetine, in fact, had to be titrated down to 7 mg daily due to activating symptoms, including the patient becoming hyperverbal. Visual hallucinations abated over time but did intermittently reoccur with new auditory and tactile hallucinations in subsequent years. The patient described hearing distressing, at times aggressive, voices but without specific commands. Tactile hallucinations included formications. Even so, his presentation was never suggestive of schizophrenia, lacking both affective blunting and characteristic negative symptoms. Risperidone was initially trialed up to 0.25 mg nightly, but the patient and family felt it had no effect, and it was discontinued. Aripiprazole was also trialed up to 3.25 mg daily but was later discontinued due to family observations of its lack of benefit. Quetiapine was later started at 100 mg daily; hallucinations decreased in frequency but did not completely resolve. The patient continues to take quetiapine. 

Concurrent with his initial therapy with fluoxetine and clonidine for anxiety, up to 0.15 mg daily was added. Outside of pharmacological management, the patient also started intense CBT with an independent psychologist. As a result, the patient was further characterized as having OCD of incompletion or "just right" OCD. This type of OCD refers to the overwhelming thoughts or worries that can occur when soothing rituals or compulsions are unable to be completed [3]. He has since undergone multiple trials of CBT, all mostly for self-limited benefit. Current symptoms have sporadically been noticeable for intermittent passive ideation of non-suicidal self-harm. At this time, the patient is presently stable, with a robust support network in his family. Worrying thoughts about new people or new family members (a newborn niece) and concerns with contamination are intermittent, but family accommodation (the act of reassurance) helps to ease patient distress. Soothing rituals (Table 2) are largely still intact.

In retrospect, the mother had noticed subclinical distressing behaviors prior to his seizure (Table 2). These behaviors included ritualized actions, walking only on white tiles, fear of contamination, difficulty self-soothing, and requiring repeated reassurances from parents. However, she concurs that the patient's behaviors considerably worsened in the days and weeks following the seizure.

Birth, developmental, social and family history

The patient was born via induced vaginal delivery at 40 weeks to an advanced maternal-age mother, age 42. Gestation was complicated by a knotted umbilical cord, a smaller stomach, and a calcified hepatic lesion.

Development was initially unremarkable, with milestones appropriately achieved through the first two years of life. Starting at two years, the patient became notable for speech delay, vocal tics, oro-motor dysfunction, and poor weight gain. The patient later received a gastric tube for nutritional supplementation, upon which he is still partially dependent, although he no longer meets the criteria for poor weight gain. The patient also continues to receive speech therapy; however, there is now less concern for oro-motor dysfunction, and tics have largely subsided. As noted above, there was an initial concern for autism spectrum disorder, but this was not definitively supported by neuropsychological testing. Testing did indicate average development of verbal and nonverbal reasoning, language functioning, attention, intact visual-spatial skills, visual-spatial integration ranging from average to impaired, and some sensory sensitivity.

At the time of the OCD diagnosis, the patient lived at home with two older siblings and both parents and maternal grandparents as caretakers. He is currently an adolescent, approximately 14 years of age, homeschooled due to social anxiety concerns for in-person school, and continues to live at home with both parents and one older sibling. Outside of his family support system, he is limited socially, but he does periodically attend online classes to help with social interaction with similar-aged peers.

Both my father and paternal grandfather have a history of epilepsy. The patient has two older siblings who have since both been independently diagnosed with OCD. The mother has no other relevant psychiatric history.

## Discussion

One limitation, in this case, is that the patient never completed testing for group A β-hemolytic streptococcal or GABHS-induced autoantibodies. However, other autoimmune workups were negative. These antibodies are part of the routine workup for rapid-onset OCD and would support a diagnosis of PANDAS. The patient and their family were unable to complete testing due to insurance coverage, personal reluctance, and because multiple specialists in psychiatry, psychology, and neurology from geographically separate health systems were being simultaneously consulted. That being so, this may not be problematic because there are several reasons why the patient was less suspicious of PANDAS.

Not only did the family deny any historical symptoms consistent with a strep pharyngitis infection in the months preceding his OCD diagnosis, but PANDAS has an episodic course where obsessive-compulsive or OC symptoms will relapse, remit, and be exacerbated by repeat GABHS infections [9-11]. Our patient (Table 2) had OC symptoms going back months and years before being formally diagnosed with OCD. OC symptoms never remitted following his seizure but rather increasingly worsened. Indeed, retrospective research has demonstrated patients will experience OC symptoms for an average of five years before meeting OCD criteria [5,9].

With the patient clinically less suspicious for PANDAS, PANS would reasonably next be considered in the workup. Historically, PANDAS was revised to be included under PANS because of the significant number of OCD patients testing negative for PANDAS autoantibodies. However, according to the diagnostic criteria, PANS is specifically excluded when OC symptoms may be reasonably ascribed to another medical or neurological disorder [7,10]. Therefore, we must consider the temporal correlation between the neurological disorder consisting of the hypoglycemic seizure (later diagnosed as epilepsy) and the exacerbation of OC symptoms.

There have been several reports of OCD exacerbated by neurological disorders. Among these is a case where an 11-year-old male previously diagnosed with OCD experienced a worsening in his symptoms following obstructive hydrocephalus secondary to glioma. Following intervention for the hydrocephalus, OC symptoms only temporarily improved [12]. The literature review also includes several cases where OCD was diagnosed following a traumatic brain injury with corroborating neuroimaging [13]. One case in particular was of a 12-year-old who was diagnosed with OCD and aggression, which resolved after two years [14].

Both cases highlight the neurophysiological underpinnings of OCD, namely the research implicating dysfunction in the cortico-striatal-thalamo-cortical (CSTC) loop [15]. In fact, dysfunction in the CSTC loop has been noted in pediatric populations even during the subclinical phase [5]. This is relevant to our patient because, in retrospect, he had many subclinical OC symptoms prior to the seizure, diagnosis of epilepsy, and OCD diagnosis.

There have long been reports correlating epilepsy with OCD, but this has mainly been with temporal lobe epilepsy (TLE). Up to 70% of patients with TLE have been diagnosed with OCD [16,17]. Although our patient was later diagnosed with epilepsy (note that he did have a supporting family history of epilepsy in his father and paternal grandfather) and did begin antiepileptic drug therapy, repeat EEGs were consistently negative, and his epilepsy was unable to be more precisely characterized. Furthermore, as mentioned above, the patient discontinued antiepileptic drug therapy after five years, as he was seizure-free between the second and fifth years following his diagnosis of epilepsy. However, his OCD persisted.

Returning to our patient’s hypoglycemic seizure, behavioral changes in the following seizures have previously been described. However, these cases have mainly been in the acute post-seizure period, with depression and anxiety symptoms typically lasting two to five days [18]. Our patient was, in fact, later diagnosed with recurrent depression and anxiety, but this was likely independent of his seizures. However, our patient also did present with hallucinations, which were initially only visual but in subsequent years did include sporadic audio and tactile hallucinations. Post-seizure psychosis, similar to post-seizure affective symptoms, tends to be more often circumscribed [19].

Our patient has had intermittent hallucinations since first being diagnosed with OCD and has partially benefited from second-generation antipsychotic therapy, only quetiapine, while having no benefit from risperidone or aripiprazole. The fact remains that his OC symptoms predate any psychotic symptoms. Moreover, although his psychotic symptoms do impact his day-to-day life (i.e., causing him distress), he has always understood them to not be real; he has no flat affect, no delusions, and no disorganized thinking. Comorbid OCD and psychosis, according to one meta-analysis, have a prevalence of anywhere from 12-24% [20]. Therefore, OCD and psychosis can and do overlap in many patients.

## Conclusions

Rapid onset and early-onset obsessive-compulsive have classically been described in the context of PANDAS/PANS. PANDAS antibodies were, unfortunately, never completed in this patient. However, a diagnosis of PANDAS was less likely, as shown above. Although PANS allows for acute stressors other than GABHS, the criteria exclude neurological or medical disorders. Therefore, a diagnosis of rapid-onset OCD with hallucinations in the context of a discrete neurophysiological stressor, i.e., a seizure, represents a unique clinical presentation of pediatric obsessive-compulsive disorder. The uniqueness of this case is highlighted by the fact that although comorbid OCD and epilepsy cases have been previously described in other literature, to the best of our knowledge, this is the first observation wherein the acute stressor might reasonably be due to a seizure. It is our hope that this case report will contribute to a body of novel presentations of OCD with implications for the neurodevelopment of other pediatric patients with similar psychopathologies. It is also our hope that this case report will contribute to the ever-growing body of literature describing new psychiatric symptomatology in post-seizure patients.
